# Response to RET-Specific Therapy in *RET* Fusion-Positive Anaplastic Thyroid Carcinoma

**DOI:** 10.1089/thy.2019.0477

**Published:** 2020-09-08

**Authors:** Dora Dias-Santagata, Jochen K. Lennerz, Peter M. Sadow, Ryan P. Frazier, Sandya Govinda Raju, Dahlia Henry, Trisha Chung, Jennifer Kherani, S. Michael Rothenberg, Lori J. Wirth

**Affiliations:** ^1^ Department of Pathology, Massachusetts General Hospital, Boston, Massachusetts, USA.; ^2^ Loxo Oncology, Inc., Stamford, Connecticut, USA.; ^3^ Department of Medicine, Massachusetts General Hospital, Boston, Massachusetts, USA.

**Keywords:** anaplastic thyroid carcinoma, gene fusion, LOXO-292, *RET*, tumor genomic profiling

## Abstract

***Background:*** Anaplastic thyroid carcinoma (ATC) remains one of the most challenging malignancies to treat in the modern era. Most patients present with or develop recurrent/metastatic incurable disease with poor response rates to conventional chemotherapy, and life expectancy is short. Next-generation sequencing (NGS) can be leveraged in ATC to identify oncogenic alterations that can be targeted with molecularly specific therapy, offering new effective treatment options to a subset of patients.

***Patient Findings:*** A 73-year-old man presenting with locally advanced papillary thyroid carcinoma containing a minor component of ATC was treated with surgery and iodine-131. He developed biopsy-confirmed ATC distant metastases that progressed on cytotoxic chemotherapy. NGS revealed several alterations, including a *CCDC6-RET* gene fusion. The patient enrolled in LIBRETTO-001, a phase I/II trial of the potent and specific RET inhibitor, LOXO-292. The patient tolerated LOXO-292 well and experienced a deep and durable partial response, ongoing beyond 19 months.

***Conclusion:*** This clinically significant response achieved with LOXO-292 in a patient with a *CCDC6-RET* fusion-positive ATC who had exhausted conventional treatment options highlights the importance of conducting tumor genomic profiling in patients with ATC to identify uncommon but actionable genomic alterations, such as *RET* gene fusions.

## Introduction

Anaplastic thyroid carcinoma (ATC) is a rare highly aggressive malignancy that accounts for only ∼1% of thyroid cancers in the United States, but up to half of thyroid cancer deaths (1). Median disease-specific survival is only 4 months, which has remained essentially unchanged for the past few decades (2). Although intensive multimodality treatment combining surgical resection with postoperative radiation and chemotherapy can improve local control and survival for some patients with disease confined to the neck, most patients will develop progressive disease or harbor metastatic disease at presentation. Metastatic ATC is incurable and poorly responsive to conventional cytotoxic chemotherapy (3,4).

The application of next-generation sequencing (NGS) in thyroid cancer has helped define the molecular landscape of ATC, distinguished it genomically from other more well-differentiated follicular-derived thyroid cancers with a better prognosis and suggested novel targeted treatment approaches (5–7). More than half of ATCs possess activating mutations in MAPK pathway genes, including *BRAF* and the three *RAS* genes. The efficacy and safety of the BRAF inhibitor, dabrafenib, plus the MEK inhibitor, trametinib, in *BRAF^V600E^*-mutant ATC led to US Food and Drug Administration approval of this combination for *BRAF^V600E^*-mutant ATC without locoregional treatment options, the first newly approved therapy for ATC in nearly 50 years (8). The activity of dabrafenib plus trametinib in *BRAF^V600E^*-mutant ATC has established a paradigm of matching a driver genomic alteration with a specific targeted therapy in ATC and has stimulated the search for additional targetable oncogenic drivers in the majority of ATC tumors that do not harbor *BRAF* mutations.

Chromosomal rearrangements resulting in oncogenic kinase gene fusions have emerged as actionable therapeutic targets in diverse human malignancies, including chronic myelogenous leukemia and non-small cell lung cancer (NSCLC). Kinase fusions have been identified in distinct histological subtypes of thyroid cancer, including papillary thyroid cancer (PTC; *RET*, *NTRK1/2/3*), medullary thyroid cancer (*ALK)*, and poorly differentiated thyroid cancer (*RET, ALK)* (5,9,10). Although *RET* fusions were previously reported in ATCs, the samples analyzed also contained PTC tissue and/or co-occurring *BRAF* mutations. Therefore, the source of each identified *RET* gene fusion (i.e., PTC or ATC component) could not be determined (11). In recent NGS analyses of thyroid cancers, including ATCs, *RET* gene fusions were identified in more well-differentiated thyroid cancers, but were absent from ATCs (5,7).

In this study, we report a patient with ATC metastatic to multiple sites, including the brain and lungs, refractory to cytotoxic chemotherapy, with a *CCDC6-RET* fusion identified by NGS and confirmed by fluorescence *in situ* hybridization (FISH). The patient was treated with LOXO-292, a potent and highly selective RET inhibitor, in an ongoing phase I/II clinical trial. He achieved a confirmed partial response on LOXO-292, both systemically and in the brain, and remains on treatment, in response, beyond 19 months.

## Patient

A 73-year-old man presented with an asymptomatic right neck mass in June 2017. Positron emission tomography/computed tomography (CT) showed an ^18^F-fluorodeoxyglucose (FDG)-avid right thyroid mass and FDG-avid right cervical and upper mediastinal adenopathy. He underwent total thyroidectomy and bilateral neck dissection one month later at an outside hospital, where pathological evaluation revealed papillary thyroid carcinoma, predominantly diffuse sclerosing variant. Postoperatively, the patient received 150 mCi of iodine-131. His stimulated thyroglobulin was 2.1 ng/mL.

Eight months after surgery, the patient experienced mild confusion and visual disturbance attributed to partial seizure. He presented to an outside hospital emergency room, where brain magnetic resonance imaging (MRI) showed an enhancing 1.5 cm right frontal mass with surrounding edema and mild midline shift. A second enhancing 1.0 cm lesion with edema was present in the right parietal lobe. Chest CT showed new lung nodules, the largest of which was 1.8 cm, and supraclavicular adenopathy.

**FIG. 1.**
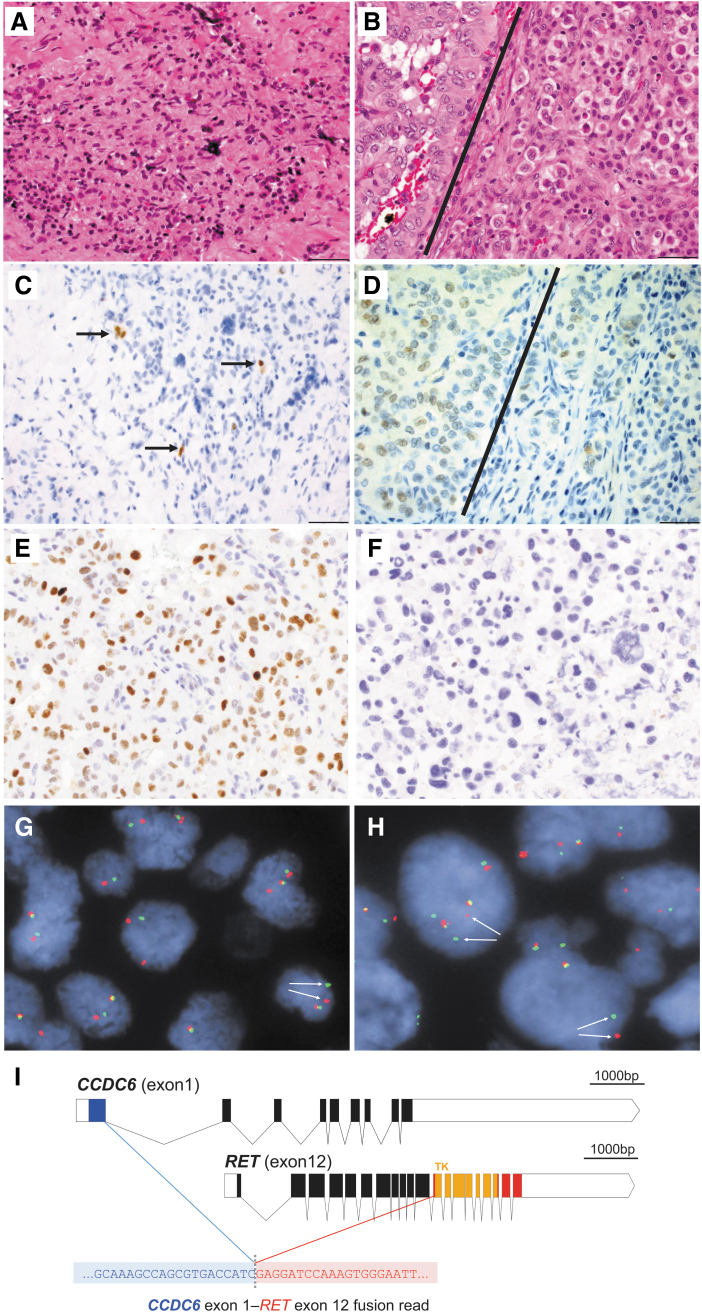
Primary thyroid tumor **(A, C)**. Lymph node metastasis **(B, D, E, G)**, resected along with the primary tumor and consisting primarily of PTC with rare atypical cells scattered among the papillary-like component **(**left **B**, left **D, E, G)** and with a minute focus of undifferentiated (anaplastic) cells **(**right **B**, right **D)**. Fine needle aspirate of a right neck metastasis consisting almost exclusively of anaplastic thyroid cancer cells **(F, H)**, collected 8 months after surgery at the time of disease progression to the brain and lungs. Hematoxylin and eosin stained photomicrographs of the primary tumor **(A)** and concurrent lymph node metastasis **(B)**. PAX8 stain of the primary tumor **(C)** and metastasis **(D)**. Decreased PAX8 stain is noted for the undifferentiated anaplastic component of the tumor **(**right **D)**. The lymph node metastasis **(B, D)** shows the more papillary-like areas **(**left of bar, **B)** have increased staining for PAX8 **(**left of bar, **D)** and the more undifferentiated (plasmacytoid/rhabdomyoblastic) areas **(**right of bar, **B)** have decreased PAX8 staining **(**right of bar, **D)**. Positive p53 immunostaining in the papillary-like component of the lymph node metastasis **(E)** resected with the primary tumor, contrasts with nearly absent p53 staining in the anaplastic thyroid carcinoma cells of the neck metastasis **(F)** detected 8 months after surgery upon disease progression. FISH analysis with break-apart *RET* probes detected the *RET* rearrangement (arrows) in both the PTC-rich component of the lymph node metastasis **(G)**, as well as in the ATC cells present in the neck metastasis **(H)** collected 8 months after surgery. 400 × magnification **(A–F)**, 600 × magnification **(G, H)**. Panel I shows the *RET* fusion transcript. Notes: fusion detection was by anchored multiplex polymerase chain reaction/RNA-Seq based on identified contiguous reads spanning from exon 1 of *CCDC6* to exon 12 of *RET*; open boxes represent 5′/3′ untranslated regions; introns are scaled to 4%. ATC, anaplastic thyroid carcinoma; bp, base pairs; FISH, fluorescence *in situ* hybridization; PTC, papillary thyroid cancer; TK, tyrosine kinase domain.

He was started on dexamethasone and levetiracetam and presented to our institution. Review of the outside surgical specimen, consisting of the primary tumor and concurrent metastasis resected in July 2017, revealed a 1.6 cm undifferentiated (anaplastic) thyroid carcinoma arising as a minor component of a PTC with oncocytic and tall cell features, exhibiting gross extrathyroidal extension and positive surgical margins (Fig. 1A, B). Seven of 30 lymph nodes were positive, with the involved nodes in the right central neck and right levels II/III and V, the largest of which was 3.0 cm with extranodal extension. The lateral neck and level V nodes had mixed features of both papillary carcinoma and undifferentiated (anaplastic) carcinoma. Immunohistochemistry staining was positive for TTF-1, keratin AE1/3/CAM5.2 and CK19, weakly positive for PAX8, present in the ATC component (Fig. 1A–D), and negative for BRAF^V600E^. Suppressed thyroglobulin was 2.6 ng/mL.

The two brain lesions were treated with stereotactic radiosurgery, 1500 cGy in one fraction. While awaiting the results of molecular testing, one cycle of doxorubicin and docetaxel was administered after radiosurgery was complete.

Molecular testing comprised DNA-based (SNAPSHOT-NGS-V2 Assay) (12) and RNA-based (Archer Solid Fusion Assay V2; ArcherDX, Inc., Boulder, CO) NGS of a mediastinal lymph node metastasis, resected along with the primary tumor. The tumor in this metastasis not only had features of a tall cell variant of PTC but also included a small population of scattered cells with atypical features (comprising 10–15% of the total tumor). NGS analysis revealed that the tumor was negative for a *BRAF* mutation, but harbored putatively pathogenic variants in *TP53* (p.Pro152TrpfsTer10; c.453_477del) and in the *TERT* promoter (C228T; c.-124C>T), and variants of uncertain clinical significance in *PTCH1* (p.Ala4Thr; c.10G>A) and *TSC2* (p.Gly1356Ser; c.4066G>A). The variants detected in this lymph node met (composed primarily of PTC with rare, scattered atypical cells) had allelic frequencies of 10–15%, consistent with subclonality. These results suggest that the mutations typically associated with a more aggressive tumor phenotype (i.e., *TP53* and *TERT*) were present in a small fraction of tumor cells, possibly in the rare population of atypical cells with high-grade features.

In addition, immunohistochemical staining demonstrated that the papillary-rich areas of this lymph node metastasis were largely positive for p53 expression (consistent with wild-type *TP53*), with occasional negative cells (Fig. 1E). By contrast, a right neck metastasis collected eight months after surgery and comprised almost exclusively of anaplastic carcinoma cells was negative for p53 IHC (consistent with mutant *TP53* gene, Fig. 1F), further suggesting that *TP53* inactivation was associated with the ATC component. Of particular note, the RNA-based NGS fusion assay showed that the PTC-rich lymph node metastasis expressed fusion transcripts derived from a *CCDC6-RET* gene fusion (Fig. 1I). FISH analysis with break-apart RET probes confirmed the presence of the RET gene rearrangement in the PTC component of the lymph node metastasis (Fig. 1G), as well as in the ATC cells of the neck metastasis detected eight months after surgery at the time of disease progression to the brain and lungs (Fig. 1H).

In May 2018, CT imaging showed disease progression after one cycle of chemotherapy, the course of which was complicated by pulmonary embolism. The patient was offered participation in the LIBRETTO-001 (NCT03157128) study and provided written informed consent at enrollment. LIBRETTO-001 is a phase I/II trial of the highly specific and potent RET inhibitor, LOXO-292; the patient received the recommended phase II dose of 160 mg orally twice daily. He tolerated LOXO-292 well, and all treatment-related adverse events he experienced were grade 1 by Common Terminology Criteria for Adverse Events (CTCAE) Version 5.

**FIG. 2.**
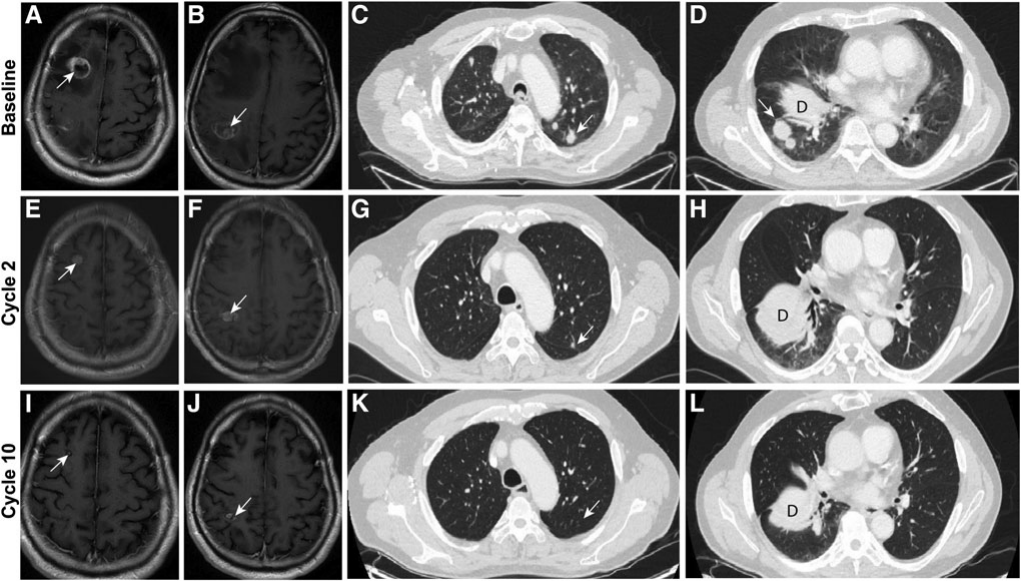
Magnetic resonance imaging of the patient's brain at baseline **(A, B)**, at the end of cycle 2 **(E, F)** and cycle 10 **(I, J)** of LOXO-292 therapy showing progressive resolution of metastatic lesions. Computed tomographic images of chest lesions before **(C, D)** and at the indicated times after initiation of LOXO-292 **(G, H, K, L)**. A radiological PR by RECIST Version 1.1 was achieved by the end of cycle 2 that deepened with continued treatment. PR, partial response.

Restaging CTs after the first 8 weeks of treatment showed a 40.0% decrease in the measurable lung nodules according to Response Evaluation Criteria in Solid Tumors (RECIST) Version 1.1 (13) (Fig. 2). This partial response was confirmed at the patient's subsequent restaging. Brain MRI showed a decrease in size of the two previously treated brain lesions (Fig. 2). The patient resumed his usual activities, including refereeing baseball games and golfing. At the time of writing, the patient remains on LOXO-292 at the full dose. His latest restaging CTs after 19 months of treatment revealed ongoing shrinkage in the measurable lesions (−56.19% from baseline by RECIST v1.1), and brain MRI showed a continued slight interval decrease in the size of the two treated metastases. The patient will remain on this clinical trial therapy until experiencing disease progression, drug intolerance, or voluntary withdrawal from the study.

## Discussion

We report an adult patient with ATC and a biopsied tumor metastasis harboring a *CCDC6-RET* gene fusion who responded dramatically to LOXO-292, a highly selective RET inhibitor currently in clinical development. *RET* gene fusions are present in ∼10–20% of PTCs (10,14) and 6% of poorly differentiated thyroid cancers (5). The incidence of *RET* gene fusions is higher in younger versus older patients with PTC, and in those with a history of radiation exposure (15–17). Although *RET* gene fusions were previously reported in ATC with mixed PTC components, it was not clear whether the ATC, PTC, or both components harbored the fusion, and *RET* fusions have not been identified in more recent ATC analyses (5,7). Our FISH analysis and NGS results indicate that, in this patient, the *RET* gene fusion was expressed in a large proportion of tumor cells in a PTC-rich lymph node metastasis and was also present in the ATC cells of a neck metastasis detected at the time of disease progression. The presence of a concomitant *TP53* mutation predominantly in the ATC component is consistent with *TP53* gene inactivation commonly observed in ATC but rarely in PTC. In summary, our molecular data together with a confirmed tumor response to the selective RET inhibitor LOXO-292, suggest that this patient's ATC was driven by the identified *CCDC6-RET* gene fusion.

The principle that tyrosine kinases activated by somatic gene fusions or point mutations are oncogenic drivers in malignancies and can be effectively targeted therapeutically, either in tumor-specific or tumor-agnostic settings is now well established (18–21). Several multitarget kinase inhibitors that target RET in conjunction with other kinases are clinically available. To date, such agents have shown only modest antitumor activity in patients with tumors harboring *RET* gene fusions, which may be a consequence of suboptimal RET inhibition due to high levels of toxicity associated with the concurrent inhibition of other kinase targets (22). In the phase I component of the LIBRETTO-001 trial investigating LOXO-292, an ATP-competitive highly selective small molecule RET inhibitor (23), LOXO-292 showed robust antitumor activity in patients with *RET* fusion-positive PTC and NSCLC, and *RET* mutation-positive medullary thyroid cancer. In addition, safety and tolerability were consistent with the highly selective drug design, with most adverse events being either grade 1 or 2, and unrelated to LOXO-292 (24–26).

ATC is a rare highly aggressive tumor with a poor prognosis (2,3). The current patient had exhausted conventional treatment options and progressed both radiographically and symptomatically. The detection of an expressed *CCDC6-RET* gene fusion in a tumor metastasis by RNA-based NGS suggested that RET inhibition might be an effective treatment option. A partial response was noted at the first response assessment after only 8 weeks of treatment with LOXO-292, which was confirmed in subsequent staging scans. The response was deep and durable, the treatment was well tolerated, and the patient was able to resume his normal activities, a remarkable outcome for a patient with metastatic ATC.

Several approaches with variable sensitivity and specificity are available to detect the presence of *RET* gene fusions to identify patients appropriate for RET inhibitor therapy, including FISH, transcript-based NanoString analysis, multiplex quantitative reverse transcription-polymerase chain reaction, and next-generation DNA- or RNA-based sequencing (NGS) (27–29). The number of described 5′ *RET* fusion partners currently exceeds 20 (22), which will likely increase as tumors are more generally profiled by NGS in research and clinical settings. The optimal diagnostic strategy to identify tumors harboring *RET* gene fusions, *RET* point mutations, or other actionable driver mutations in malignancies, including ATC, is, therefore, a broad-based NGS approach combining analysis of both tumor DNA and RNA.

In conclusion, a deep and durable partial response in a patient with ATC harboring a *CCDC6-RET* gene fusion was achieved with LOXO-292. This and the promising results from the ongoing phase I/II study of this agent highlight the importance of establishing diagnostic procedures to facilitate the routine clinical detection of somatic *RET* alterations in patients with thyroid cancer.
